# A Novel Angiotensin I-Converting Enzyme Mutation (S333W) Impairs N-Domain Enzymatic Cleavage of the Anti-Fibrotic Peptide, AcSDKP

**DOI:** 10.1371/journal.pone.0088001

**Published:** 2014-02-04

**Authors:** Sergei M. Danilov, Michael S. Wade, Sylva L. Schwager, Ross G. Douglas, Andrew B. Nesterovitch, Isolda A. Popova, Kyle D. Hogarth, Nakul Bhardwaj, David E. Schwartz, Edward D. Sturrock, Joe G. N. Garcia

**Affiliations:** 1 Institute for Personalized Respiratory Medicine, University of Illinois at Chicago, Chicago, Illinois, United States of America; 2 Department of Anesthesiology, University of Illinois at Chicago, Chicago, Illinois, United States of America; 3 Institute of Infectious Disease and Molecular Medicine and Division of Medical Biochemistry, University of Cape Town, Cape Town, South Africa; 4 Department of Dermatology, Rush University, Chicago, Illinois, United States of America; 5 Chemistry of Life Processes Institute, Northwestern University, Evanston, Illinois, United States of America; 6 Department of Medicine, University of Chicago, Chicago, Illinois, United States of America; 7 Department of Medicine, University of Arizona, Tucson, Arizona, United States of America; Max-Delbrück Center for Molecular Medicine (MDC), Germany

## Abstract

**Background:**

Angiotensin I-converting enzyme (ACE) has two functional N- and C-domain active centers that display differences in the metabolism of biologically-active peptides including the hemoregulatory tetrapeptide, Ac-SDKP, hydrolysed preferentially by the N domain active center. Elevated Ac-SDKP concentrations are associated with reduced tissue fibrosis.

**Results:**

We identified a patient of African descent exhibiting unusual blood ACE kinetics with reduced relative hydrolysis of two synthetic ACE substrates (ZPHL/HHL ratio) suggestive of the ACE N domain center inactivation. Inhibition of blood ACE activity by anti-catalytic mAbs and ACE inhibitors and conformational fingerprint of blood ACE suggested overall conformational changes in the ACE molecule and sequencing identified Ser333Trp substitution in the N domain of ACE. *In silico* analysis demonstrated S333W localized in the S_1_ pocket of the active site of the N domain with the bulky Trp adversely affecting binding of ACE substrates due to steric hindrance. Expression of mutant ACE (S333W) in CHO cells confirmed altered kinetic properties of mutant ACE and conformational changes in the N domain. Further, the S333W mutant displayed decreased ability (5-fold) to cleave the physiological substrate AcSDKP compared to wild-type ACE.

**Conclusions and Significance:**

A novel Ser333Trp ACE mutation results in dramatic changes in ACE kinetic properties and lowered clearance of Ac-SDKP. Individuals with this mutation (likely with significantly increased levels of the hemoregulatory tetrapeptide in blood and tissues), may confer protection against fibrosis.

## Introduction

Angiotensin I-converting enzyme (ACE, CD143) is a Zn^2+^ carboxydipeptidase which plays a key role in the regulation of blood pressure and also in the development of vascular pathologies and tissue remodeling. ACE is constitutively expressed on the surface of endothelial cells, epithelial, neuroepithelial and cells of immune system (macrophages and dendritic cells) as a membrane-bound protein and has been designated as CD143. Two homologous domains (N and C domains) comprise the majority of the structure of somatic ACE (sACE) and each contain a functional Zn^2+^ binding active center reviewed in [1]–[2].

The three-dimensional crystal structure of sACE is still unknown. However, the model of the two-domain ACE has been recently suggested [3]–[4] based on the solved crystal structures of the C and N domains [5]–[6], epitope mapping of mAbs to ACE [7], and, on the electron microscopy picture of sACE [3].

Several mutations in ACE have been described: with several producing familial elevation of blood ACE due to effects on the rate of ACE shedding such as P1199L [8]–[11], Y465D [4], and R532W [12]. In contrast, other ACE mutations abolish transmembrane anchoring to cell membrane resulting in direct ACE secretion into the blood, i.e. W1197X [13], IVS25+1G>A [14]. Finally, yet other ACE mutations such as transport – defective ACE mutation - Q1069R [15] and likely many others [16] impaired trafficking to the cell surface and caused renal tubular dysgenesis due to almost complete absence of catalytically ACE on the cell surface.

We now report a novel ACE mutation, where residue substitution (S333W) near the active site, altered kinetic characteristics of the N domain active center of somatic ACE. Mutated ACE exhibited decreased hydrolysis of physiological, N domain specific substrate Ac-SDKP, a negative regulator of the hematopoiesis [17] with strong anti-fibrotic properties [18]. Potential clinical consequences of this mutation may confer protection from lung fibrosis [19].

## Experimental Procedures

### Study participants

The study was approved by the Institutional Review Boards of the University of Chicago and the University of Illinois at Chicago. Participants provided their written informed consent to participate in this study. The IRBs approved this consent procedure. Serum ACE levels were assessed in serum present in the University of Chicago Biobank obtained from patients with different sarcoidosis phenotypes (along with controls). One patient (#27) was found to have serum ACE with an unusual kinetic characteristics –low (0.7) ZPHL/HHL ratio - normal value -1.05±0.05 [20].

### ACE activity assay

ACE activity in serum/plasma or culture fluids or lysates from ACE-expressing cells was measured using a fluorimetric assay with two ACE substrates (2 mM Z-Phe-His-Leu or 5 mM Hip-His-Leu [21]–[22], respectively. Briefly, 40 µl aliquots of samples diluted in PBS-BSA (0.1 mg/ml), were added to 200 µl of ACE substrate and incubated for the appropriate time at 37°C. The His-Leu product was quantified fluorometrically, via complexing with ortho-phtaldialdehyde. Determination of the ratio of hydrolysis of the two substrates (ZPHL/HHL) was performed as described [20]. In some experiments samples containing any sources of WT or mutant ACEs were pre-incubated during 1 hour with different ACE inhibitors (1 µM) or anti-ACE mAbs (10 µg/ml).

### Immunological characterization of the mutant ACE

Ninety six-well plates (Corning, Corning, NY) were coated with anti-ACE mAbs [23] and incubated with serum/plasma samples or medium/lysates of CHO-ACE expressing cells After washing of unbound ACE, plate-bound ACE activity was measured by adding a substrate for ACE directly into wells [24].

### Sequencing and genotyping

Genomic DNA was obtained from whole blood of patient # 27 and 13 exons of ACE gene (6th–12^th^, 19^th^–24th) were sequenced, using primers designed by [25], obtained from SeqWright, Inc. (Houston, TX) and designed in-house.

### Molecular modeling of ACE

Analysis of the N domain was based on the recently published [6] crystal structure (PDB accession number 3NXQ) and the C domain was based on the structure resolved by Natesh et al. [7] (PDB accession number 1O86). PYMOL (http://www.pymol.org) was used to make the S333W substitution *in silico* using PyMol Mutagenesis Wizard.

### Site-directed mutagenesis and in vitro analysis of the mutant ACEs

cDNAs encoding mutant ACE proteins were created by GenScript USA Inc. (Piscataway, NJ) by mutation of the 1) T**C**G codon for Ser at position 333 (somatic mature ACE numbering [26] to codon TGG for Trp and 2) **G**AT codon for Asp at position 562 to codon GGT, in expression vector based on pcDNA3.1+/Hygro (Invitrogen Corp., Carlsbad, CA and containing the full-length somatic ACE cDNA controlled by CMV early promoter [27].

Plasmids carrying the coding sequence for wild-type ACE and above mutants were expressed in CHO cells using Plus Reagent (Invitrogen Corp., Carlsbad, CA) for transient transfection and generation of stable cell lines. Serum-free culture medium from these cells was used as a source of the secreted (soluble) ACE (wild type and mutants) for biochemical and immunological characterization. Lysate of these cells obtained with detergent Triton X-100 (0.5% in PBS) was used as a source of membrane-bound form of WT and mutant ACE.

### Western blot analysis of mutant ACE expression

Lysates from CHO-WT-ACE cells (with ACE activity of 5 mU/ml using Hip-His-Leu) were compared to lysates from CHO-ACE–S333W cells normalized by equal protein loading by SDS-PAGE in 4–15% acrylamide Tris-HCl pre-cast SDS PAGE gels (Bio-Red Laboratories, Hercules, CA). After electrophoretic transfer of proteins to microporous PVDF-Plus membrane Western blotting was performed exactly as in [4].

### Kinetic assessment of AcSDKP hydrolysis by recombinant ACE

An adapted plate assay was designed based on previously published approach for *N*-acetyl-angiotensin I [28] using fluorescamine (4-phenylspiro[furan-2(*3H*),1′-phthalan]-3,3′-dione), a reagent that forms fluorescent adducts exclusively with primary amines [29]. 30 µl of 1 mM AcSDKP (Sigma-Aldrich Co.) substrate in buffer (50 mM HEPES, pH 7.5, 100 mM NaCl, 10 µM ZnSO_4_) was warmed to 37°C in a 96-well plate. The assay was commenced by the addition of 10 µl (10 µg total protein) pre-warmed lysate, incubated for 30 minutes and stopped by the addition of 50 µl 1 M HCl. The solution was neutralized by the addition of 50 µl 1 M NaOH and the pH increased to 8.3 by the addition of 100 µl 500 mM K_2_HPO_4_/KH_2_PO_4_ buffer pH 8.3. 10 µl of fluorescamine (2 mg/ml in acetone, Sigma-Aldrich Co.) was added and the resulting mixture incubated for 3 minutes at room temperature. Fluorescence intensities were measured at λ_ex_ = 390 nm and λ_em_ = 475 nm using a Cary Eclipse spectrofluorimeter (Varian Inc.). Changes in fluorescence compared to empty vector lysate were converted to reaction velocities by the use of a standard curve. The standard was constructed by complete hydrolysis of various concentrations of AcSDKP by purified N-domain using the same approach as above.

## Results and Discussion

### Identification of novel ACE mutations

**Figure 1:**
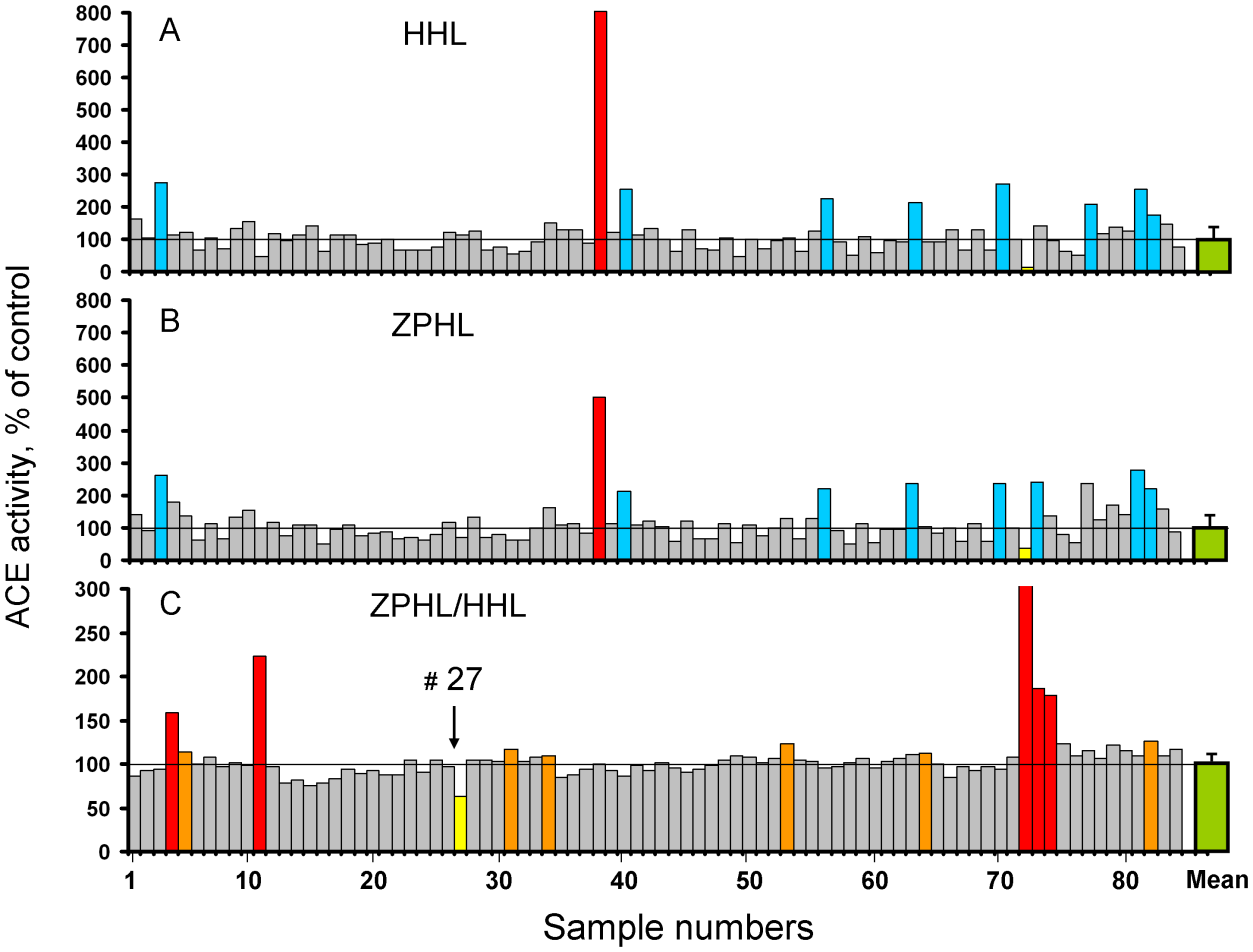
ACE activity in human serum. **A–B**. ACE activity in 85 samples of human serum from patients with different pulmonary diseases and unrelated patients (served as a controls) - TRIDOM study, was quantified using a spectrofluorometric assay with Hip-His-Leu (A-5 mM) and Z-Phe-His-Leu (B-2 mM) as substrates. Data expressed as % of individual ACE's activity from mean value for whole population (33.0 mU/ml with Hip-His-Leu). Bars highlighted with blue - samples with values of ACE activity higher than mean + 2SD. Bar highlighted with yellow-sample # 72 with very low ACE activity –less than 30% of mean value. Bar highlighted with red-putative ACE mutation. C. Ratio of the rate of hydrolysis of the two substrates (ZPHL/HHL ratio) in the tested samples. Data expressed as % of individual ZPHL/HHL ratio from mean value for whole population (1.05+0.05). Calculation of this parameter was used primarily for identification of patients with ACE inhibitors in their blood (20): highlighted by red (high concentration) and orange (low, but detectable concentration of ACE inhibitors). Bar highlighted by yellow (and arrowed)-from patient # 27. The results are shown as mean values of duplicates. (Values of standard deviations, which were not exceeded 10%, were not shown for clarity).

Screening of serum ACE activity was performed in 84 cases with pulmonary and extrathoracic sarcoidosis and unrelated patients serving as controls and resulted in the identification of a single case (#38) with ACE activity 5-fold higher than controls (Fig. 1A and 1B). Further investigation led to the discovery that this patient carried a novel ACE mutation-R532W [12]. ACE activity in serum of 84 patients was determined with two artificial “short” substrates HHL and ZPHL (Fig. 1A and 1B). Fig. 1C shows the ratio of the rate of hydrolysis of two ACE substrates, ZFHL and HHL (ZPHL/HHL ratio), which was used primarily to detect presence of ACE inhibitors in the blood of patients at the time of blood sampling [20]. Eleven patients (red and orange bars in Fig. 1C) had elevated ZPHL/HHL ratios suggesting the presence of an ACE inhibitor. However, one patient (#27) demonstrated a markedly lower ZPHL/HHL ratio of 0.7. This represents the lowest ZPHL/HHL ratio we have determined in more than 600 plasma/serum samples (not shown).

The two domains of ACE hydrolyze a range of natural and synthetic substrates, but with different efficiencies [30]–[33]. The two synthetic substrates ZPHL and HHL are used for determination of ACE activity in laboratories worldwide. These two substrates display some contrasting enzymatic properties: the C domain of human ACE hydrolyzes HHL at a much faster rate (9–fold) than the N domain [30], whereas ZPHL is hydrolyzed at a similar rate by both domains [23]. As a result, the ratio of the rates of hydrolysis of these two substrates (ZPHL/HHL ratio) is characteristic of different forms of ACE: somatic (two-domain) human ACE - about 1-1.5, N-domain – 5–7, and C-domain - 0.6–0.8 [20]. The selective inactivation or inhibition of the C-domain in somatic ACE increases this ratio to higher values more characteristic for the N-domain, whereas selective inactivation/inhibition of the N-domain in somatic ACE decreases the ratio toward lower values predicted for C-domain [20].

**Figure 2:**
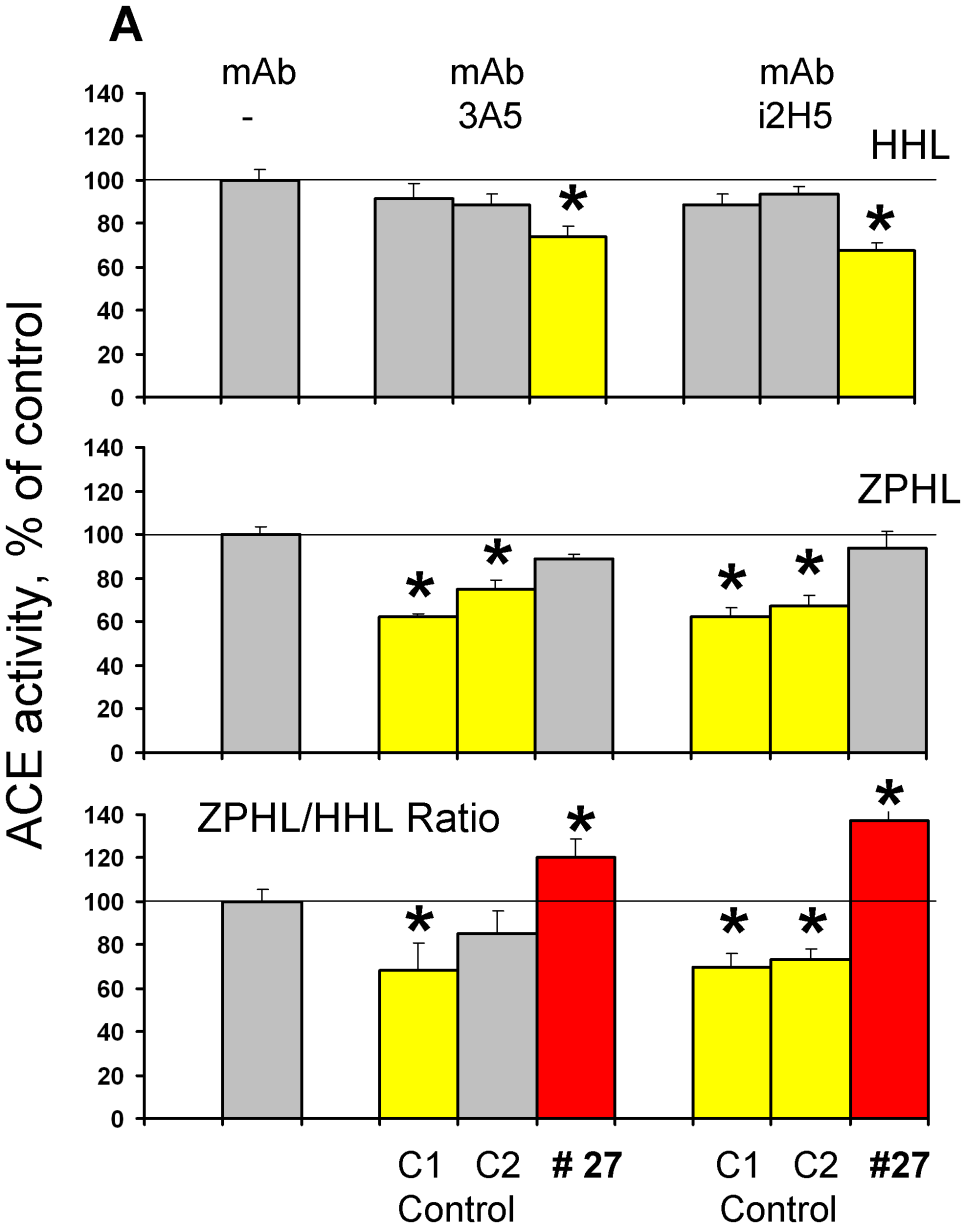
Effect of of anti-catalytic mAbs to N-domain active center for A and C for B) and from patient # 27 (arrowed in Fig. 1 C) were incubated with mAbs which are anti-catalytic for the N-domain active center - i2H5 and 3A5 (10 µg/ml)[23], [34]. Residual ACE activity was determined with two ACE substrates as in Fig. 1. We also measured ZPHL/HHL ratio-as in [20] and in Fig. 1 C. Bars highlighted with yellow - samples with values of ACE activity lower than mean ± 2SD for control sample without mAbs. Bars highlighted with red- samples with values of ZPHL/HHL ratio higher than mean ± 2SD for control samples without mAbs. Data presented are mean ± SD of 2–3 independent experiments in duplicates). * p<0.05 vs. control serum.

**Figure 3:**
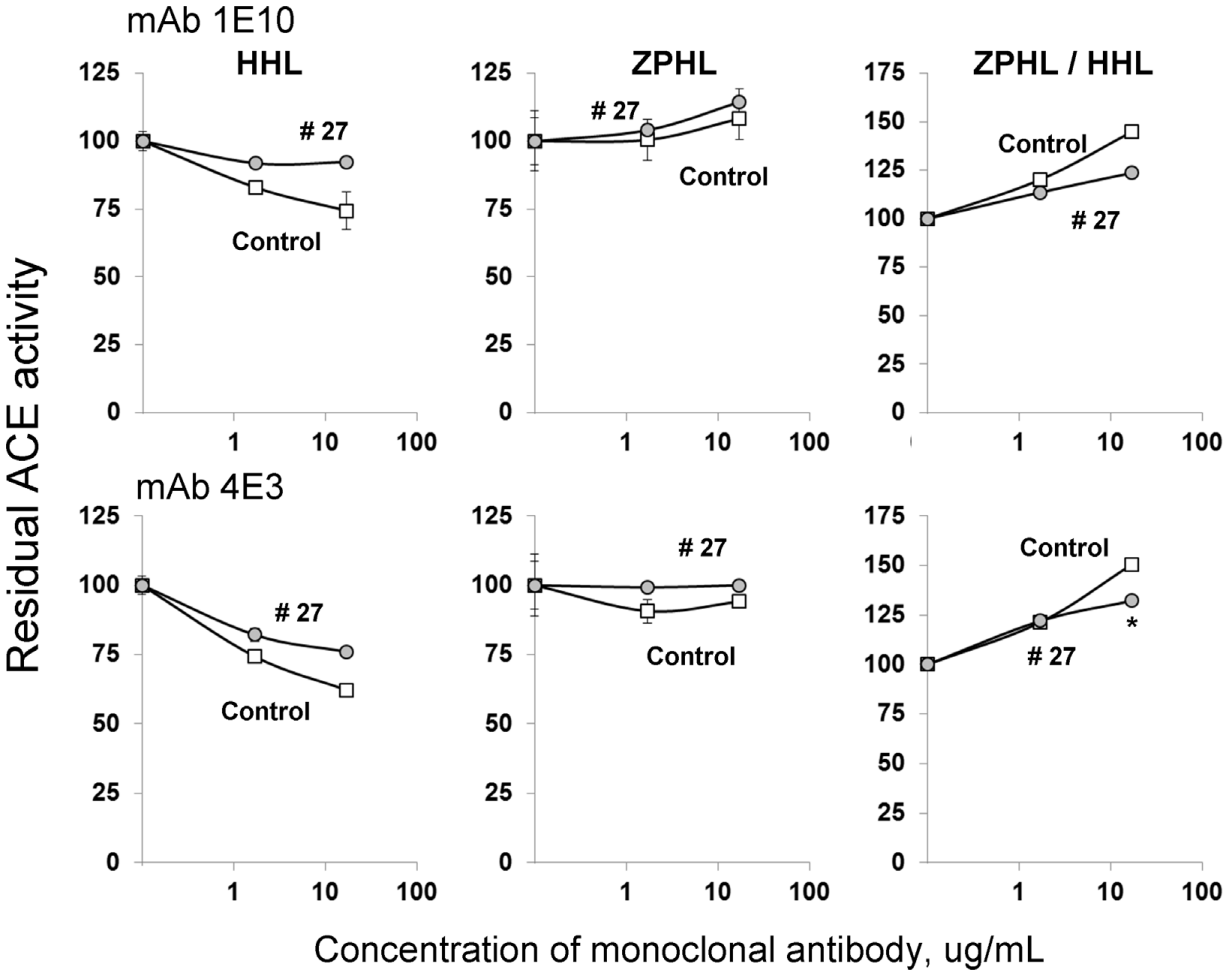
Effect of of anti-catalytic mAbsto C domain active center on blood ACE activity. Serum samples from pools of healthy volunteers (Control) and from patient # 27 (arrowed in Fig. 1 C) were incubated with mAbs which are anti-catalytic for the C -domain active center –1E10 and 4E3 [7]. Residual ACE activity was determined with two ACE substrates as in Fig. 1. We also measured ZPHL/HHL ratio-as in [20] and in Fig.1 C Data presented are mean ± SD of 2–3 independent experiments in duplicates). * p<0.05 vs. control serum.

Therefore, the lower value of ZPHL/HHL ratio for patient # 27 suggested that the N domain active center could be inactivated by a mutation in the active site of the enzyme. To further investigate this, we incubated serum from patient # 27 (and two controls - healthy individuals with normal ZPHL/HHL ratio) with mAbs 3A5 and i2H5 which are anti-catalytic towards the N-domain active center [23], [34]. The inhibition of ACE activity from patient # 27 by the anti-catalytic mAbs 3A5 and i2H5 was weaker with substrate ZPHL than HHL (Fig. 2). This was surprising, considering the substrate HHL is preferentially hydrolyzed by the C-domain and the binding of anti-catalytic mAbs to the epitopes localized on the N domain essentially did not affect the hydrolysis of HHL by healthy individuals (controls) (Fig. 3). Moreover, mAbs 1E10 and 4E3 were less effective in the inhibition of ACE activity with HHL than with ZPHL.

**Figure 4:**
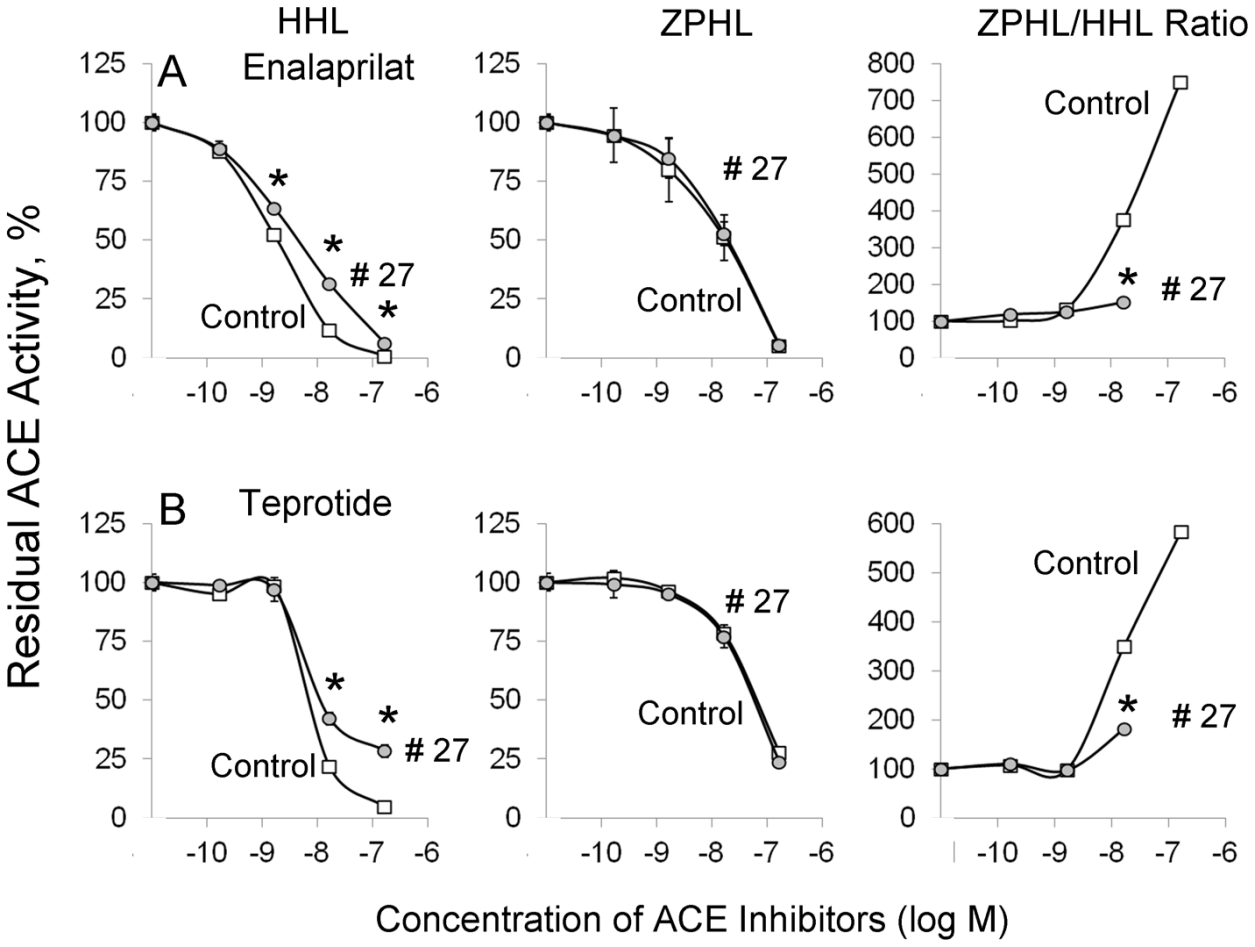
Effect of ACE inhibitors on blood ACE activity. Serum samples (1/5 dilution in PBS) from pool of 10 healthy volunteers (control) and from patient # 27 (arrowed in Fig. 1 C) were incubated with different concentration of ACE inhibitors: short –enalaprilat (**A**) and long –teprotide (**B**). Residual ACE activity was determined with two ACE substrates (and ZPHL/HHL ratio) as in Fig. 2. Data presented are mean ± SD of 2–3 independent experiments in duplicates). * p<0.05 vs. control serum.

The pattern of inhibition of blood ACE activity from patient #27 was similar for the short tripeptide ACE inhibitor enalaprilat (Fig 4A) and long nonapeptide ACE inhibitor teprotide (Fig. 4B) using substrate ZPHL. However, inhibition by these inhibitors using substrate HHL was less effective: IC_50_ for enalaprilat was 2 nM for control serum and 7 nM for serum from patient # 27 (p<0.05); for teprotide IC_50_ values were 9.5 nM and 13.0 nM, respectively (p<0.05). Thus, the data presented in Fig. 2–4 may indicate that the putative ACE mutation in patient # 27 could affect the overall catalytic ability, which can change the relative ability to hydrolyse tested substrates rather than just inactivation of the N-domain active center.

In order to characterize the conformation of blood ACE from patient #27 we performed conformational fingerprinting of blood ACE, using a panel of mAbs directed against 16 different epitopes located on the N-and C-domain of catalytically active human ACE [24]. We recently demonstrated that the pattern of precipitation of ACE activity – (conformational fingerprint of ACE) using a set of mAbs directed against 16 different epitopes of human ACE provides a sensitive means of identifying changes in local conformation of ACE due to inactivation, inhibition, mutations, etc. [24]. Moreover, we successfully used this set of mAbs to detect several new human ACE mutations and characterized the conformation of Pro1199Leu [35], Trp1197Stop [13], Q1069R [15], Y465D [4] ACE mutations.

**Figure 5:**
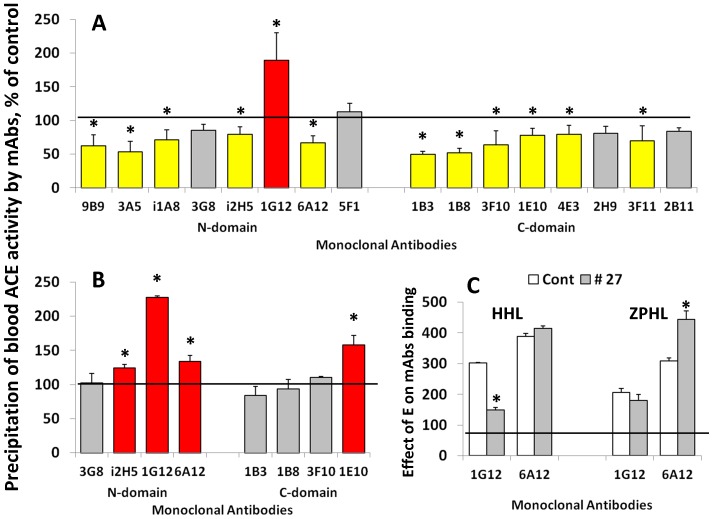
Conformational fingerprinting of serum ACEs with a set of mAbs to ACE. **A**. Sixteen monoclonal antibodies were used to precipitate ACE from sera. Immunoprecipitated ACE activity is presented as a normalized value (“binding ratio”), to highlight differences in immunoprecipitation pattern (“conformational fingerprint”) among blood ACE from patient # 27 and blood ACE from healthy individuals (which were expressed as 100%). **B**. Binding of several mAbs to serum ACE from control samples and serum from patient # 27-relative to binding by mAb 9B9. Data expressed as % of mAbs/9B9 binding ratio for serum from patient # 27 to that for control serum. Bars highlighted with yellow - samples from serum of patient # 27 with values of precipitated ACE activity 20% lower than this value for control sample. Bars highlighted with red- samples from serum of patient # 27 with values of precipitated ACE activity 20% higher than this value for control sample. **C**. Effect of ACE inhibitor enalaprilat (100 nM) on binding of mAbs 1G12 and 6A12 to serum ACE from control samples and serum from patient # 27. All data presented as a mean of 3–5 independent determinations. * p<0.05 indicates values shown is significantly different.

The immunoprecipitation profile of plasma ACE from subject # 27 is quite different to that of plasma ACE from healthy volunteers with normal ZPHL/HHL ratio (Fig. 5A). The binding of 11 of the 16 mAbs ACE from this patient decreased, while binding of mAb 1G12 and mAb 1E10 increased significantly. This suggests that there is a change in overall conformation of ACE from patient #27. We also estimated local conformational changes of blood ACE from this patient, by calculating the relative binding of mAbs to different epitopes on the N- and C-terminal domain. Binding of several mAbs, sensitive to local conformational changes in ACE (due to denaturation, glycosylation, and inhibitor binding) [24], [36] relative to binding of mAb 9B9, directed to the epitopes resistant to such changes [37] is shown in Fig. 5B. These results confirmed that the region on the surface of the N domain of ACE containing overlapping epitopes for mAbs 1G12 and 6A12 and the region on the C domain of ACE containing epitope for mAb 1E10, undergoes a significant conformational change.

We demonstrated previously that binding of mAbs 1G12 and 6A12 to blood ACE dramatically (3–4-fold) increased in the presence of ACE inhibitors [36] ACE inhibitor enalaprilat increased binding of mAb 6A12 to ACE from control and patient # 27 to a similar extent, whereas increase in mAb 1G12 binding to ACE from patient # 27 was lower to normal ACE from control blood. Moreover, this difference was apparent only with substrate Hip-His-Leu and not with Z-Phe-His-Leu (Fig. 5C). This suggests that conformational changes in blood ACE of patient # 27 could be very localized proximal to the region where Hip-His-Leu binds to the N-domain active site.

Therefore fine analysis of enzymatic activity, sensitivity to ACE inhibition and conformational analysis of blood ACE from patient # 27 (Figures 1–5) unequivocally demonstrated profound local and overall conformational changes in ACE from this patient, which likely altered the interactions of ACE from this patient with, at least, artificial ACE substrates.

We sequenced those exons of the ACE gene from this patient which code amino acid residues comprising the N- and C-domain active sites [26], or contribute to N-and C-domain substrate specificity [38] - 6th–12^th^ exon of the N domain and 19th–23th exon of the C domain. We identified several known polymorphic variants of ACE gene in patient # 27: i) rs 12709426 (c.1809A>G; p.D592G), which correspond to D563G substitution - mature somatic ACE numbering [26] in 12^th^ exon of ACE; ii) rs12720737 in intron 23; and iii) rs4362 (c.3421T>C, synonymous substitution in 1129 codon for Phe).

**Figure 6:**
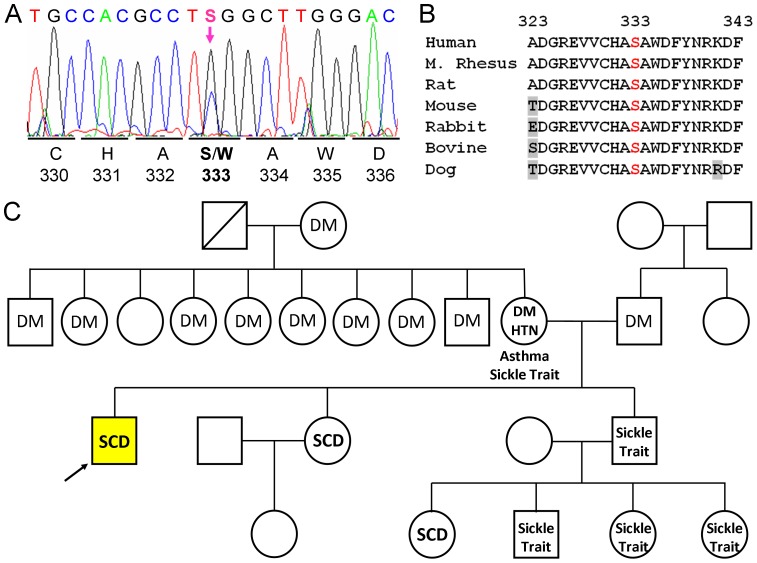
Identification of novel ACE mutation- Ser333Trp. **A**. Sequencing of ACE gene in patient # 27. Heterozygous mutation Ser333Trp (S333W) in 7^th^ exon was revealed by the sequencing 6–12^th^, 19^th^–24th exons of ACE gene of this patient. **B**. Alignment of amino acid sequences from ACE of several vertebrate species indicates that S333 and the flanking residues are highly conserved. The numbering relates to the mature ACE. **C**. Family tree of patient # 27 with new (S333W) mutation in ACE gene. Genotyped individual with S333W mutation are indicated by yellow color. Individuals found to have low value of ZPHL/HHL ratio for serum ACE are marked by upward pointing arrows. Following abbreviations are used for known clinical diagnoses: SCD-Sickle Cell Disease, DM–diabetes mellitus, HTN–hypertension. Among individuals in whom both genotyping and serum ACE determination was performed, there was 100% concordance between presence or absence of the S333W mutation and low or non-elevated ZPHL/HHL ratio for serum ACE, respectively.

A heterozygous mutation was found in the 7^th^ exon c.1119 C>G, p.S362W (Fig. 6A). Additional screening revealed that none of the 100 randomly chosen genomic DNA samples from healthy controls showed this mutation indicating that this substitution is not a polymorphism. This mutation results in the substitution of a highly conserved serine (Fig. 6B) with a tryptophan, which corresponds to a S333W substitution in the N-domain of somatic ACE. Unfortunately, other members of patient's family (Fig. 6C), were not available for analysis.

The mutation in the same codon of ACE coding for Ser333 of mature ACE was found previously in at least six individuals in North America (GenBank – rs142328237, http://www.ncbi.nlm.nih.gov/projects/SNP/). Besides, the S333W substitution, an S333L substitution was found the GenBank in the same codon which should have a lesser effect on the N domain active center than the S333W substitution, due to the smaller size of the Leu side chain, compared to Trp.

Functioning of ACE as an enzyme is not tolerated by this (S362W) amino acid change (S333W for mature ACE protein) according to Polyphen-2 analysis (http://genetics.bwh.harvard.edu/pph2/, based on [39] which predicts that this mutation is damaging with a probability 100%. SIFT software [http://sift.jcvi.org/based on [40] also predicts damaging effect of this mutation: SIFT Score: 0, Median Information Content: 2.4.

**Figure 7:**
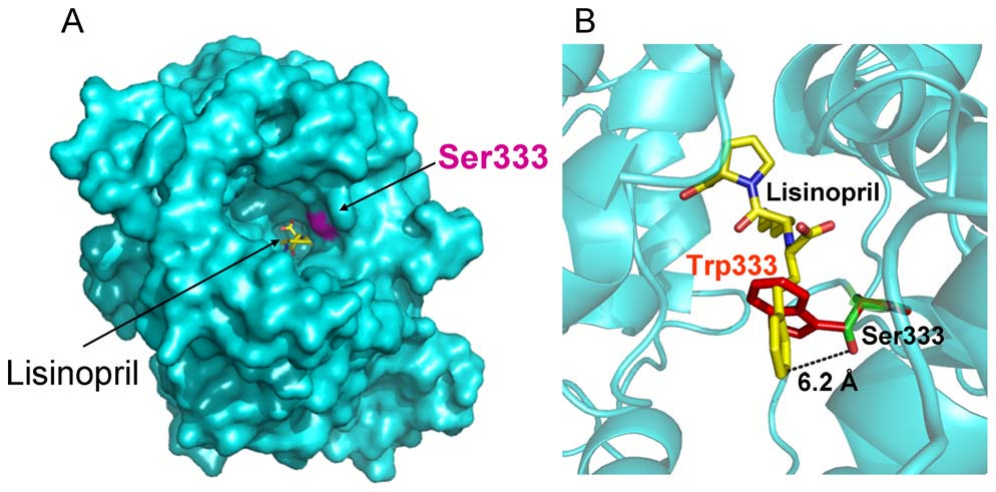
Localization of ACE mutation (S333W) in the N domain of human ACE. **A**. A molecular surface representation of the N-domain crystal structure (PDB code 2C6N [6] showing the active-site channel and the bound lisinopril (yellow stick representation). The surface of Ser333 is shown in purple. Helices α1, α2 and α3 that form a cover over the central channel have been deleted to visualize the inhibitor and the location of Ser 333. **B**. Details of the active site of a ribbon diagram of the N-domain where Ser333 has been replaced with a Trp (red stick representation) using PyMOL Mutagenesis Wizard (DeLano Scientific, Palo Alto, CA, U.S.A). Ser333 is located 6.2 Å from the P1 Phe of the ACE inhibitor lisinopril shown in yellow. Whereas Trp333 could potentially result in a steric clash with the P1 residue of the bound ligand.

**Figure 8:**
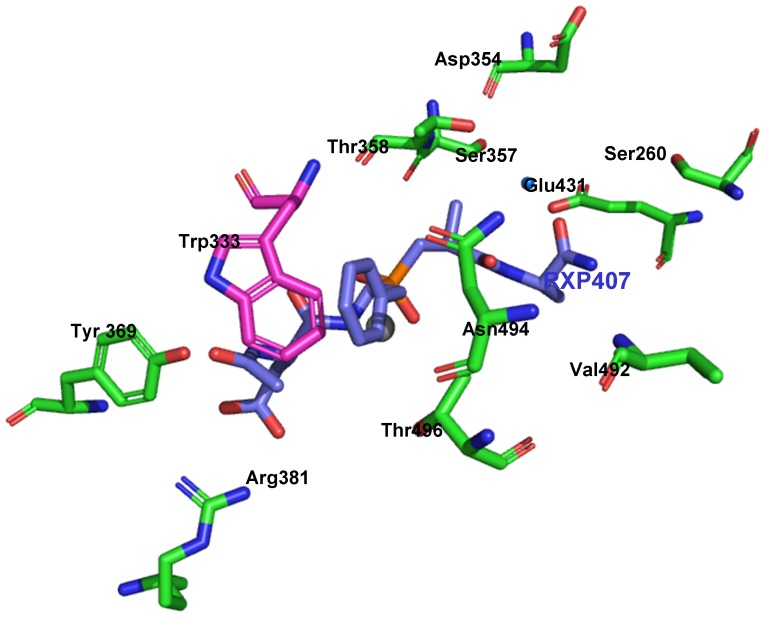
Critical residues in the active center of the N-domain of mutant ACE. The RXP407–N-domain co-crystal structure of the N domain of ACE with N-domain specific inhibitor RXP407 - PDB accession code 3NXQ [42]. Key amino acids in the N domain crucial for substrate specificity and ACE inhibition are denoted by somatic ACE numbering and are shown as green sticks. The catalytic zinc ion is shown in slate blue and RXP407 in blue. A water molecule that could possibly be involved in an interaction between the C-terminal amide of RXP407 and Thr358 is given as a small blue sphere. Image generated using PyMOL software and Ser333 has been replaced with a Trp (purple stick representation) using PyMOL Mutagenesis Wizard (v 0.99, DeLano Scientific).

Analysis of the 3D-structure of the N domain, co-crystallized with lisinopril, demonstrated that Ser333 is located deep in the active site cleft close to lisinopril (Fig. 7A). Closer inspection of the structure of the N-domain active center showed that the S333W substitution could be responsible for the dramatic decrease in the ZPHL/HHL ratio of the mutant ACE. The distance between S333 and the P_1_ phenylalanine of the ACE inhibitor lisinopril of about 6.2 Å is too far for a direct interaction with substrate, or interference with the residues in the active center responsible for substrate specificity or domain specificity of ACE inhibitors (Fig. 7B). However, if the Trp is substituted for Ser333, rotation about the side chain torsions *χ*1 and *χ*2 can result in a steric clash with the P_1_ Phe of lisinopril. Similarly, with a more extended ligand such as the N-domain selective phosphinic peptide inhibitor RXP407, it is likely that the influence of steric hindrance of the P_1_ group with Trp333 would adversely affect its binding to the active site (Fig. 8). Thus, these molecular models suggest that substitution of Ser333 by Trp could be responsible for the changes in the substrate specificity that we observed with blood ACE of the patient # 27.

### Characterization of S333W ACE Mutant

To elucidate the mechanism by which S333W substitution (or D563G) might be responsible for the changes in enzyme activity of this blood (and perhaps tissue) ACE in the affected individuals, we performed site-directed mutagenesis of human recombinant somatic ACE. These mutants of somatic ACE were expressed in CHO cells and the biochemical and immunological characteristics of these mutants were compared with WT somatic ACE.

**Figure 9:**
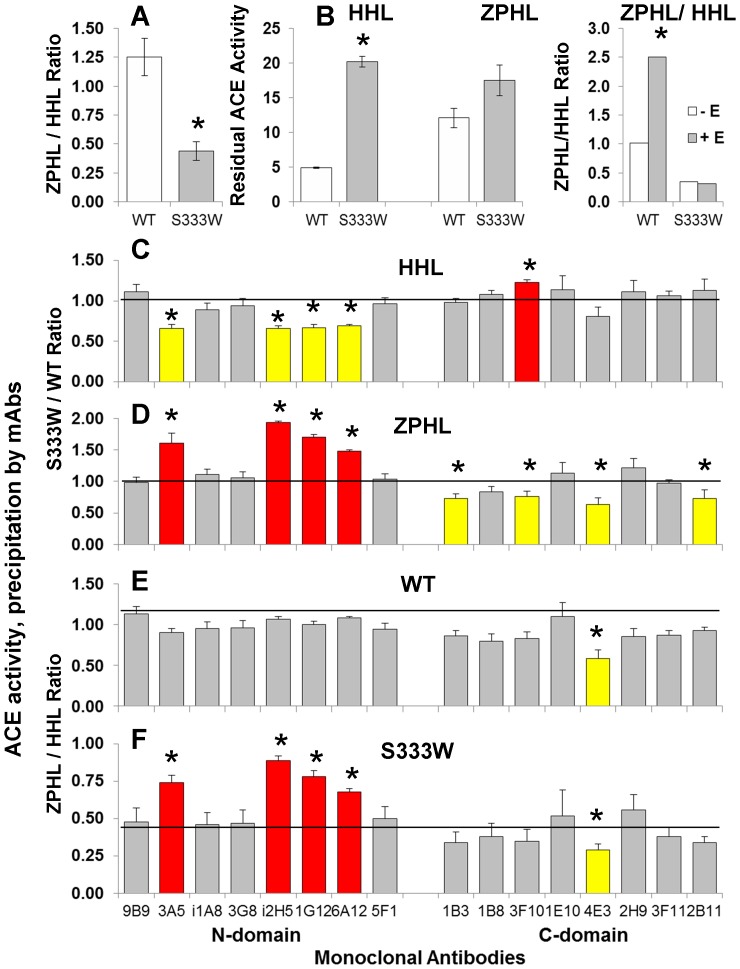
Characterization of recombinant mutant (S333W) ACE. **A**. ACE activity of the membrane-bound form of ACE of CHO cells (lysates) transiently expressing WT and mutant ACE was determined as in Fig. 1. Ratio of hydrolysis of the two substrates (ZPHL/HHL ratio) in the tested samples are presented as mean (±SD) from 4 independent experiments. *p<0.05 vs. WT. **B**. Effect of ACE inhibitors and anti-catalytic mAbs on mutant ACE activity. Lysates form CHO cells expressing mutant and WT membrane-bound ACE were incubated with ACE inhibitor enalaprilat (100 nM). Residual ACE activity was determined with both substrates and ZPHL/HHL ratio was determined as in A. **C–F**. Conformational fingerprinting of mutant ACE. **C–D**. Membrane-bound WT and mutant ACE (lysates from corresponding CHO cells) were normalized to achieve 5 mU/ml ACE activity with Hip-His-Leu as substrate and incubated in microtiter plate wells covered with 16 mAbs to human ACE as in Fig. 4 Data (mean ± SD of 2–3 independent experiments in duplicate) are expressed as ratio of ACE activity with HHL (C) and ZPHL (D) precipitated by mAbs from mutant ACE to that of WT ACE. Bars highlighted with yellow – the values of precipitated ACE activity from mutant ACE was 20% lower than this value for WT. Bars highlighted with red- the values of precipitated ACE activity from mutant ACE was 20% higher than this value for WT. **E–F**. The data presented in C and D were expressed as the ZPHL/HHL ratio of ACE activity precipitated by each mAb. Data are mean ± SD of 6–8 independent experiments in duplicate. Bars highlighted with yellow – the values of ZPHL/HHL ratio of the precipitated ACE activity was 20% lower than this value for this type of ACE in solution. Bars highlighted with red - the values of ZPHL/HHL ratio of precipitated ACE activity was 20% higher than this value for this type of ACE in solution. * - p<0.05 vs. ZPHL/HHL ratio for corresponding values of WT and mutant ACE in solution (horizontal lines in E and F). The ratio for any duplicate samples of WT ACE or mutant ACE for each mAb was approximately 1.0 and the SD was less than 10%.

As expected, based on published crystal structures of the N domains with ACE inhibitors [6], [41], D563G substitution did not cause any changes in kinetic properties of recombinant mutant ACE. In contrast, the recombinant mutant (S333W) expressed in CHO cells exhibited a dramatic decrease in ZPHL/HHL ratio (Fig. 9A) when compared to wild-type enzyme. The 65% decrease of ZPHL/HHL ratio for recombinant S333W ACE was significantly higher than the 33% we observed for blood ACE from patient #27 (Fig. 1C). This could be explained by the fact that blood of patient #27 contains a mixture of WT and mutant alleles of ACE, and thus the apparent ZPHL/HHL ratio of ACE determined in plasma of this patient is not an accurate reflection of this parameter. Mutant recombinant membrane-bound ACE also demonstrated lower sensitivity towards ACE inhibitor enalaprilat with substrate Hip-His-Leu than towards Z-Phe-His-Leu (Fig. 9B), similar to what we observed with plasma ACE from patient # 27 (Fig. 4). Therefore, these data unequivocally demonstrate that the heterozygous S333W substitution found in ACE of patient # 27 is largely responsible for the altered enzymatic activity of blood ACE, and, likely, tissue ACE in this patient.

We also performed conformational fingerprinting of recombinant S333W ACE (Fig. 9C–E) in the same way as we did for plasma ACE from patient #27 (Fig. 5). The conformational fingerprint of mutant ACE (i.e. the pattern of binding of 16 mAbs to ACE) was quite different to that seen for WT ACE, supporting the hypothesis that a single amino acid substitution can cause overall conformational changes in the ACE molecule as well as local conformational changes close to this residue. However these changes were greatly dependent on the type of ACE and on ACE substrates used.

The overall precipitation of ACE activity (measured with Hip-His-Leu as a substrate) from membrane-bound mutant ACE by mAbs directed to the epitopes localized on the N-domain was lower than that by mAbs to C-domain. The mean S333W/WT ratio of ACE activity precipitation for 8 mAbs to the N domain calculated from the data presented in Fig.9C was 0.79±0.13 versus 1.06±0.11 (p<0.05) for mAbs directed to C domain, and, especially, binding of mAbs i2H5, 1G12 and 6A12 whose epitopes overlap [23], [36] decreased significantly. The pattern of overall precipitation of ACE activity from membrane-bound mutant ACE, measured with ZPHL was quite different from that measured with HHL, but also differs between N and C domain mAbs. Furthermore, the changes due to the S333W mutation were more prominent for mAbs having overlapping epitopes– i2H5, 1G12,and 6A12 (Fig. 9D).

These data suggest that the S333W substitution leads to local conformational changes in the N-domain of the mutant ACE (selective, local denaturation of the N domain of ACE) which then leads to changes in the hydrolysis of some substrates.

We also assessed the ZPHL/HHL hydrolysis by mutant ACE bound to antibodies. The ZPHL/HHL ratio of WT ACE bound to mAbs did not change significantly except for mAb 4E3 which is strongly anti-catalytic for the C domain active center [7]. However, the putative selective denaturation of the N domain of mutant ACE was partially restored after binding to those mAbs which showed impaired binding (Fig. 9C). The ZPHL/HHL ratios for the mutant ACE precipitated by mAbs to the C-domain was the same as that for mutant ACE in solution, whereas the ZPHL/HHL ratio for mutant ACE bound to mAbs 3A5, i2H5, 1G12 and 6A12 t significantly increased, demonstrating a shift towards higher values characteristic of native two-domain ACE (Fig.9E). We already observed a local chaperone-like effect of mAbs to the C-domain of ACE directed to the regions close to a mutation in the C-domain - Q1069R [15].

**Figure 10:**
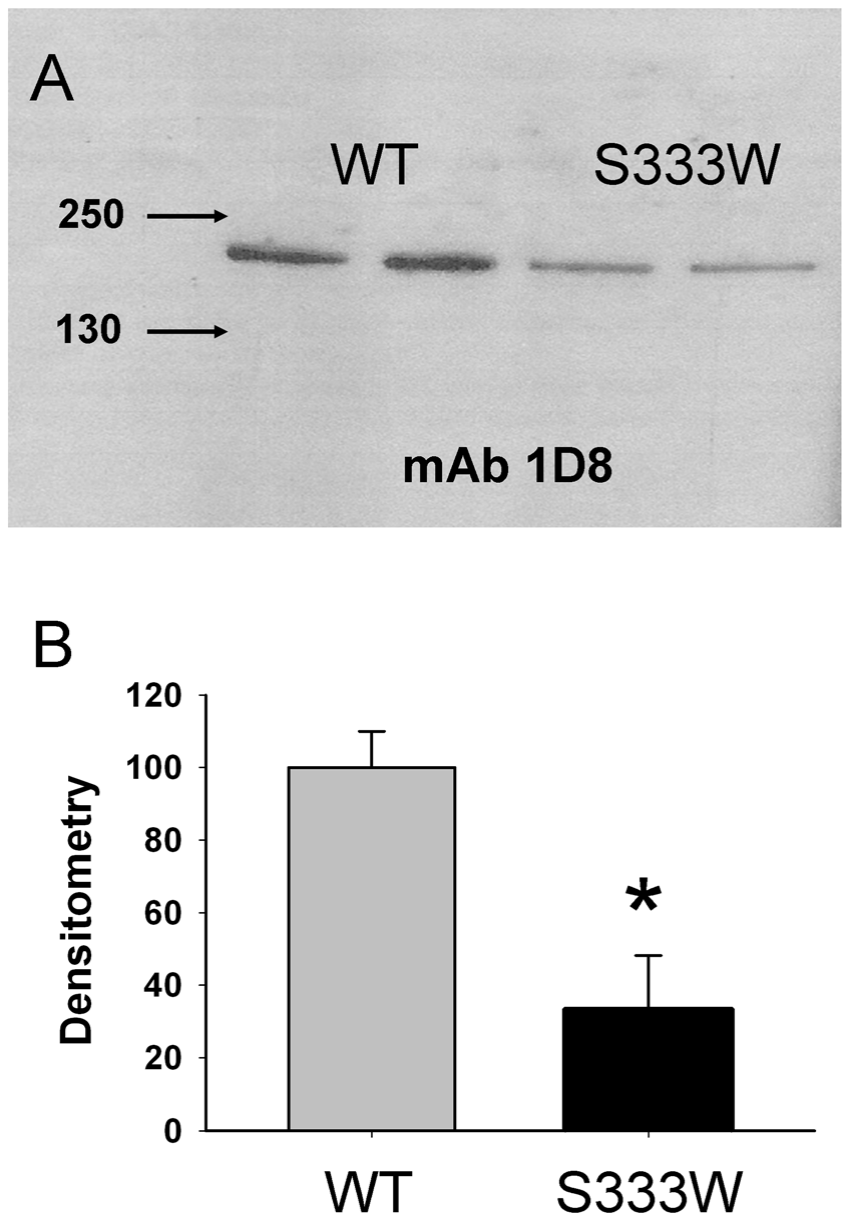
Mutant ACE protein quantification (Western Blotting). CHO cells were transiently transfected with plasmids coding for wild-type (WT) and mutant (S333W) ACE (4 µg of plasmid DNA per 35 mm dish). The lysates of these cells (normalized by equal protein loading of 10 µg per lane) were subjected to SDS-PAGE (4–15% gradient gel) in reducing conditions for Western blotting (**A**) or protein quantification (**B**). **A**. Western blotting was performed with mouse mAb 1D8 that recognizes the denatured epitope on the C-domain of human ACE [45]. Proteins transferred on PVDF-Plus membrane were revealed with 1/5 dilution of culture medium from hybridoma cells producing mAb 1D8. Molecular weight markers are shown by arrows on the left of panel A, which is a representative experiment. **B**. The relative amount of WT and mutant ACE revealed by Western blotting with mAb 1D8 (**A**) was quantified by the image analysis (densitometry) using ImageJ software (NIH). Data are expressed as mean ± SD of 2 independent measurements.

We also performed Western blotting of cell lysates from CHO-WT-ACE and CHO-S333W-ACE using mAb to denatured human ACE -1D8 which recognize sequential epitope in the C domain [42]. Western blot of recombinant wild-type and membrane-bound mutant ACE with mAb 1D8 demonstrates the amount of mutant ACE protein loaded for electrophoresis was three times lower than the amount of wild-type ACE (Fig. 10), despite the fact that ACE activity of both proteins was equilibrated with Hip-His-Leu. Bearing in mind that the ACE activity in mutant protein determined with substrate Z-Phe-His-Leu was 3-fold less, it is likely that mutant ACE hydrolysed substrate Hip-His-Leu roughly 3-fold faster than wild type N domain.

Our data indicates that dramatic changes in enzymatic activity of mutant S333W ACE (ZPHL/HHL ratio) could be due to two reasons: 1) the presence of a bulky Trp in the active site cleft that might affect the binding of ACE substrates due to steric hindrance; and 2) selective denaturation of the N-domain of mutant ACE, perhaps due to incorrect folding and trafficking to the cell surface.

We demonstrated that this mutation (S333W) abolished substrate specificity of the N-domain at least for two artificial substrates Hip-His-Leu and Z-Phe-His-Leu. The N-terminal domain of WT human ACE cleaves Hip-His-Leu much slower than the C-domain [23], [30], whereas the N-domain active center cleaves substrates such as LH-RH and AcSDKP much more efficiently [31], [43]. Several active site residues determine the substrate specificity of the N- and C-domains active centers of somatic ACE [15], [38].

**Figure 11:**
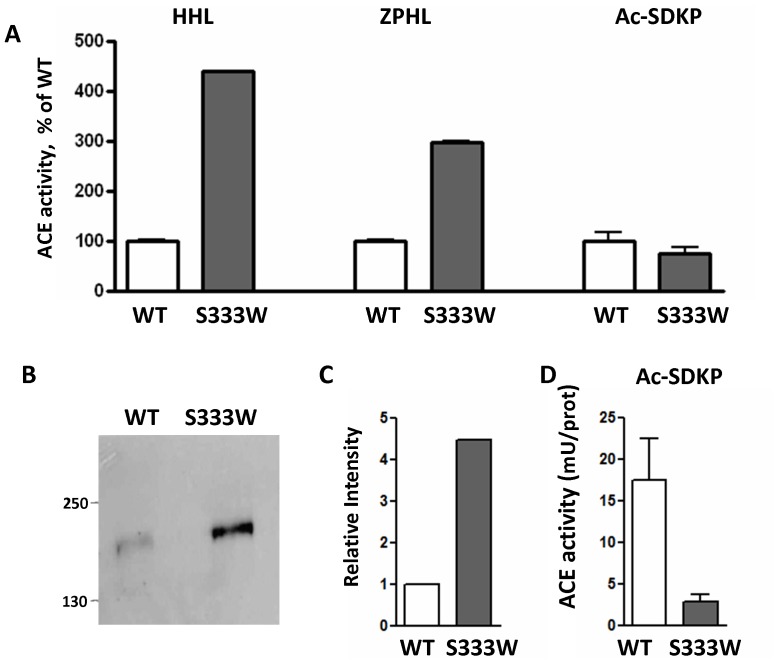
Ac-SDKP hydrolysis by recombinant mutant (S333W) ACE. **A**. ACE activity of the membrane-bound form of ACE of CHO cells (lysates) stably expressing WT and mutant ACE was determined with substrates Hip-His-Leu and Z-Phe-His –Leu (as in Fig. 8) and with natural substrate Ac-SDKP (see Experimental Procedures). ACE activity (mU/ml of the lysate) was expressed as % of WT. **B–C**. The lysates of these cells (normalized by loading of 10 µg protein per lane) were subjected to SDS-PAGE (4-15% gradient gel) in reducing conditions for Western blotting (**B**) or protein quantification (**C**). **B**. Western blotting was performed with rat mAb 4G6 that recognizes the denatured epitope on the C-domain of mouse and human ACE [50]. Proteins transferred on PVDF-Plus membrane were revealed with 1/5 dilution of culture medium from hybridoma cells producing mAb 4G6. Molecular weight markers are shown by arrows on the left of panel B, which is a representative experiment. **C**. The relative amount of WT and mutant ACE revealed by Western blotting with mAb 4G6 (**B**) was quantified by the image analysis (densitometry) using ImageJ software (NIH). Data are expressed as mean ± SD of 2 independent measurements. **D**. ACE activity in the lysates of CHO cells stably transfected with WT and mutant ACE towards natural substrate Ac-SDKP 0.75 mM (A) was normalized to the amount of ACE protein in the lysate, determined by densitometry (C) after Western blotting (B).

Therefore we tested the effect of the S333W mutation on the hydrolysis of the natural substrate AcSDKP (Fig. 11). Stable transfection of CHO cells with WT- and S333W-ACE revealed a similar decrease in enzymatic activity for the mutant ACE to that seen with transient transfections using substrates HHL and ZPHL. Hydrolysis of the N-domain selective substrate AcSDKP by S333W was approximately 5-fold lower than that of WT ACE (Fig. 11D). These data suggests that patients with the S333W mutation might have increased endogenous levels of AcSDKP. (Unfortunately, repeat blood/urine samples from this patient as well as blood samples from the other 6 individuals from the NCBI data base were not available for further analysis). Nevertheless we believe that the carriers of this mutation might demonstrate decreased predisposition to certain types of fibrosis due to increased levels of the anti-fibrotic AcSDKP.

The rising burden of pulmonary fibrosis in the U.S. currently accounts for more than 40 000 deaths annually. Moreover, few effective drugs are presently available for the treatment of pulmonary fibrosis. Activation of the renin-angiotensin system (RAS) and production of Angiotensin II is associated with tissue fibrosis, therefore ACE inhibition and/or AII antagonism has been suggested as a possible anti-fibrotic therapy -see for review [18].

AcSDKP is a natural inhibitor of hematopoietic stem cell proliferation [17] and natural substrate for N-domain active center of ACE [43] has recently emerged as an anti-fibrosis molecule-for review see [18], [44]. Several *in vivo* studies demonstrated that AcSDKP injection [45]–[47] or specific prevention of AcSDKP degradation [19] may suppress fibrosis in several animal models. ACE is the primary enzyme responsible for the degradation of AcSDKP and its actions in producing the profibrotic molecule angiotensin II and in degrading the anti-fibrotic molecule AcSDKP suggest that ACE and the RAS might be important in some fibrotic diseases [19]. Anti-fibrotic effect of AcSDKP are likely mediated via prevention of TGFβ-induced Smad2 phosphorylation-for review see [18].

The inhibition of ACE in both mice and humans causes a 5-fold increase in AcSDKP levels [48]. A similar increase in AcSDKP is observed in ACE-null mice and mice with enzymatically inactive N-domain [49]. The carriers of S333W mutation in the active center of the N domain of ACE (where almost exclusive hydrolysis of AcSDKP occurs) could be protected to some extent from AcSDKP-dependent lung (and other tissues) fibrosis.

In summary, we identified a patient with a novel mutation of ACE-S333W. *In silico* analysis demonstrated that this mutation was localized in the S_1_ pocket of the active site of the N domain and it is likely the bulky Trp (W) would affect the binding of ACE substrates due to steric hindrance. We studied the effect of this mutation on the hydrolysis of several artificial and natural substrates were determined. Mutant ACE (S333W) expressed in CHO cells decreased significantly the hydrolysis of N-domain specific substrates, including the anti-fibrotic Ac-SDKP. Besides, expression of mutant ACE in CHO cells allowed us to study immunoreactivity, conformation, rate of ACE shedding and trafficking to the cell surface for the mutant ACE. Using a novel conformational fingerprinting approach for ACE, we demonstrated that S333W mutation lead to conformational changes in whole ACE, and specifically (preferentially) in the N-domain. Therefore, this study provides important insights into the mechanism by which the novel ACE mutation (S333R) results in changed kinetic characteristics of mutant ACE.
